# The COVID-19 pandemic: broad partnerships for the rapid scale up of innovative virtual approaches for capacity building and credible information dissemination in Africa

**DOI:** 10.11604/pamj.2020.37.255.23787

**Published:** 2020-11-19

**Authors:** Ambrose Otau Talisuna, Boukare Bonkoungou, Fausta Shakiwa Mosha, Bruce Baird Struminger, Jutta Lehmer, Sanjeev Arora, Ishata Nannie Conteh, John Adabie Appiah, Jeremy Nel, Shaheen Mehtar, Janet Victoria Diaz, Marta Lado, Christian Boyd Ramers, Kevin Babila Ousman, Peter Gaturuku, Alexandre Tiendrebeogo, Richard Mihigo, Zabulon Yoti, Francis Chisaka Kasolo, Joseph Waogodo Cabore, Matshidiso Rebecca Moeti

**Affiliations:** 1 World Health Organization, Regional Office for Africa, Brazzaville, Republic of Congo,; 2 World Health Organization, Intercountry Support Team, Harare, Zimbabwe,; 3 University of New Mexico ECHO Institute, New Mexico, USA,; 4 Paediatric Critical Care, Ghana,; 5 University of the Witwatersrand, Johannesburg, South Africa,; 6 Infection Control Africa Network, Cape Town, South Africa,; 7 University of Stellenbosch, Cape Town, South Africa,; 8 World Health Organization, Headquarters, Geneva, Switzerland,; 9 Partners in Health, Sierra Leone,; 10 Laura Rodríguez Research Institute, Family Health Centers of San Diego, San Diego, USA,; 11 University of California, San Diego School of Medicine, California, USA

**Keywords:** COVID-19, outbreak, public health emergency, epidemic, international health regulations, pandemic, virtual training, World Health Organization, Africa

## Abstract

The Corona Virus Disease 2019 (COVID-19) pandemic has rapidly spread in Africa, with a total of 474,592 confirmed cases by 11^th^ July 2020. Consequently, all policy makers and health workers urgently need to be trained and to access the most credible information to contain and mitigate its impact. While the need for rapid training and information dissemination has increased, most of Africa is implementing public health social and physical distancing measures. Responding to this context requires broad partnerships and innovative virtual approaches to disseminate new insights, share best practices, and create networked communities of practice for all teach, and all learn. The World Health Organization (WHO)-Africa region, in collaboration with the Extension for Community Health Outcome (ECHO) Institute at the University of New Mexico Health Sciences Center (UNM HSC), the West Africa college of nurses and the East Central and Southern Africa college of physicians, private professional associations, academia and other partners has embarked on a virtual training programme to support the containment of COVID-19. Between 1^st^ April 2020 and 10^th^ July 2020, about 7,500 diverse health professionals from 172 locations in 58 countries were trained in 15 sessions. Participants were from diverse institutions including: central ministries of health, WHO country offices, provincial and district hospitals and private medical practitioners. A range of critical COVID-19 preparedness and response interventions have been reviewed and discussed. There is a high demand for credible information from credible sources about COVID-19. To mitigate the “epidemic of misinformation” partnerships for virtual trainings and information dissemination leveraging existing learning platforms and networks across Africa will augment preparedness and response to COVID-19.

## Introduction

On the 31^st^ December 2019 the People’s Republic of China reported a pneumonia of unknown cause from Wuhan, China [1]. Within months, the epidemiological situation rapidly evolved and the virus, that we now call SARS-CoV-2, had spread to all continents, including the WHO Africa region. As of 12^th^ July 2020, 12.5 million confirmed cases, including 570,000 deaths had been reported globally [2].

Due to the rapid evolution of the outbreak, the World Health Organization (WHO) Director-General (DG) convened the International Health Regulations (2005) Emergency Committee (EC) on the 30^th^ January 2020 and declared COVID-19 a Public Health Emergency of International Concern (PHEIC) [3,4]. On the 11^th^ of March WHO declared COVID-19 a pandemic [5]. There has been rapid spread of COVID-19 in Africa, with a total of 474,592, confirmed cases and 8,210 deaths reported by 11^th^ July 2020 Africa [6]. Being a novel virus, its epidemiology is evolving, and lessons are being learned in real time. Building on the progress achieved to strengthen preparedness to mitigate risk of potential public health emergencies in the WHO Africa region [7-9], it is urgent for all countries to remain vigilant and to continue to develop capacity to prevent, detect, investigate and respond quickly to any COVID-19 cases.

As the COVID-19 pandemic evolves all countries in Africa have reported cases [6]. Consequently, all health workers in Africa urgently need to be trained and to have access to the most current evidence-based information to prevent and to manage any cases of COVID-19. While the need for rapid training has increased, most countries have instituted measures for Nonpharmacological Interventions (NPI) including stay at home orders, physical distancing, border closures, cancellation of in person meetings and 14 days quarantine after travel to areas with COVID-19 transmission [10]. These measures have gravely hampered the usual training methods for disseminating best practices and building capacity of health workers in the WHO Africa region. To respond to the COVID-19 pandemic, innovative approaches leveraging virtual capacity building and training are needed to disseminate new insights, share best practices, and create networked communities of practice for all teach, and all learn.

In view of these challenges, the WHO Africa region, in collaboration with the ECHO Institute at the University of New Mexico Health Sciences Center (UNM HSC), the West Africa college of nurses and the East Central and Southern Africa college of physicians has adapted the Extension for Community Healthcare Outcomes (ECHO) programme to support the containment of the COVID-19 pandemic [11,12]. The programme is a collaborative distance education and tele-mentoring model for health worker training and care management empowering clinicians and public health professionals to strengthen public health programs through rapid dissemination of evidence-based best practices, and WHO guidelines, and strengthen the quality of clinical care to more people, right where they live. It is a case-based low dose, high frequency educational intervention which strengthens knowledge, clinical and public-health practice through a combination of videoconferencing [to bridge geography], case-based learning, review of published literature, promotion of best practices, and monitoring and evaluation of outcomes to improve program quality [13,14]. The programme is aimed at rapidly disseminating up to date information and best practices, building capacity, and improving communication, coordination, and collaboration for COVID-19 preparedness and response among public health leaders, regional, national, and local government agencies, healthcare professionals, and community stakeholders.

The objectives of the programme are to: 1) strengthen health workers´ knowledge and share best practices for COVID-19 preparedness and response; 2) improve health workers´ comprehension of critical pillars for COVID-19 preparedness and response; 3) create a variety of communities of practice targeting different health worker cadres; 4) ensure timely dissemination of new COVID-19 guidelines and information to all health workers; and 5) create a virtual communication and learning platform to promote both South-to-South collaboration within Africa and North-to-South experience sharing.

## Seminar

### Methods

**Program description:** the diverse target audience for the programme includes professional associations and their memberships working at primary, secondary and tertiary level facilities as well as the private sector. Policy makers, doctors, nurses, allied staff, and health facility administrators all participate in the programme learning together in an interprofessional manner. Prior to the stay at home orders, the WHO Africa region had developed a package of training materials for each of the nine critical pillars of COVID-19 preparedness and response designed for implementation through in person training. These training packages have been adapted for use in various virtual web-based platforms to ensure the rapid dissemination of relevant information that is appropriate and has the right visual metaphor. Careful consideration has been given to ensure that participants have access to the resources they need. Where internet bandwidth is limited, WHO is exploring alternative approaches for information dissemination including video clips, text chat, collaborative white boarding, fun memes and reducing the resolution of images to decrease “lag time” problems. In addition, a mobile participant attestation application (app) which will document participation and store and allow sharing of all training resources is being developed.

**Figure 1:**
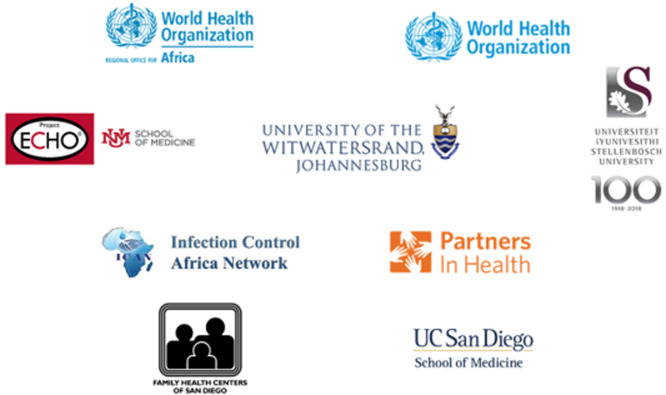
subject matter experts drawn from WHO, universities, technical agencies and other partners in the region and sub region

**Management structure:** strategic coordination team: there is a strategic coordination team that is directing this COVID-19 capacity building initiative and participates in routine virtual planning calls and is composed of the WHO regional office, Project ECHO, the WHO emergency hubs, and the secretariat of the professional associations and other key regional stakeholders to discuss stakeholder/partner engagement, program design, monitoring and evaluation. Subject matter experts and the technical team: subject matter experts are drawn from WHO, universities, and technical agencies and other partners in the region and sub regions (Figure 1). The technical teams are responsible for providing the training and responding to key questions from participants. A capacity building technical officer is responsible for coordinating the activities of each session, facilitating or co-facilitating remote participation during group discussions, and tracking outcomes, including attendance.

### Results

**Figure 2:**
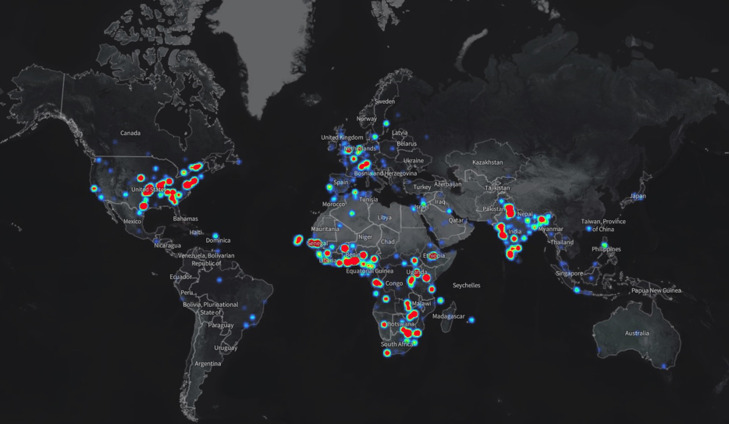
geographical information system map showing the location of participants, globally for session 4

**Figure 3:**
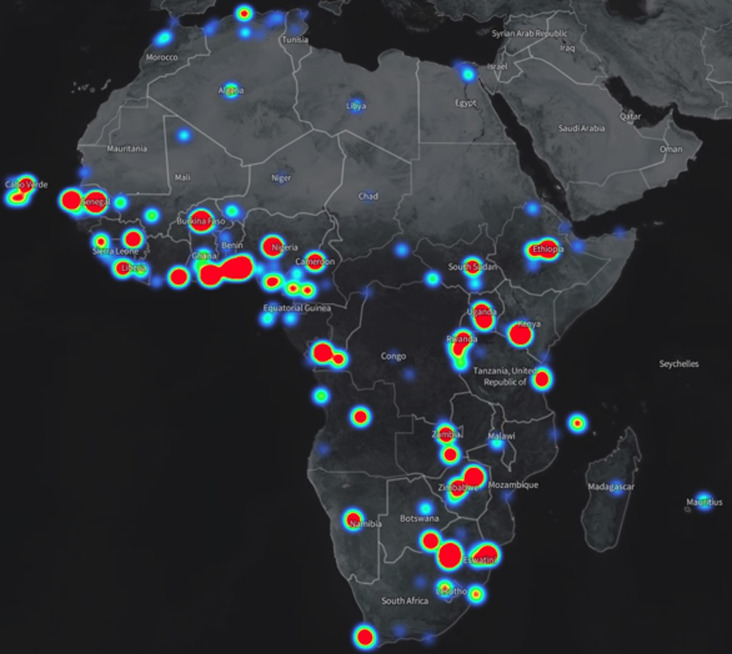
geographical information system map showing the location of participants from Africa for session 4

Between 1^st^ April and 10^th^ July, 2020, fifteen sessions have been held. The inaugural virtual session for COVID-19 was held on the 1^st^ of April 2020 focusing on the clinical management of severe acute respiratory illness (SARI) associated with COVID-19. Five hundred participants from many countries across Africa were trained. The second session conducted over 2 days was on COVID-19 surveillance, laboratory diagnosis, case management and infection prevention and control, and the target audience was all the 47 WHO country offices, the West Africa College of Nurses (WACN) and the East Central and Southern Africa College of Physicians (ECSACOP). On the first day 500 health workers were trained, while on the second day 380 health workers were reached (Table 1). The third virtual learning session was dedicated to training nurses and focused on infection prevention and control delivered by subject matter experts who are nurses. During this session the WHO Regional Director gave a virtual keynote address. Four hundred and ninety eight nurses from 42 countries were trained (Table 1). Participants represented diverse institutions including central ministries of health and provincial and district hospitals. The fourth virtual learning session was conducted on April 10, 2020 focusing on various aspects of COVID-19 (Table 1). This session addressed several technical issues, including COVID-19 asymptomatic infection and transmission, the role of antimicrobial therapy, treatment of severely ill hospitalized patients, infection prevention and control, and clarified common misunderstandings between droplet and airborne transmission. Close to 7,500 diverse health professionals from 172 (towns and cities) locations in 58 countries were trained in 15 sessions (Figure 2). Most participants were from Africa (Figure 3).

**Table 1:** virtual session and topics covered

Training title	Topics covered	Target audience	Dates and duration	Language	Number of participants
Clinical Management of severe acute respiratory infections (SARI) associated with COVID-19	COVID-19 clinical characterization; Triage and hospitalization; Sepsis: Antimicrobial therapy; Treatment of the severely ill patients in facilities; Criteria of discharge and management of convalescent patients; Infection Prevention and Control; Laboratory; Quarantine and managing cases in the communities	English Speaking Countries: Clinicians, Universities,	April-1 3 hours	English	500 Many could not connect because the maximum zoom capacity had been reached
COVID-19 case management	COVID-19 Surveillance Laboratory diagnosis; Screening, Triage and Hospitalization; Infection Prevention and Control; Syndromic diagnosis of COVID-19; Co-infection-HIV, TB, influenza, Malaria, Bacteria; Isolation/Triage Design/PPE; Psychosocial support; etc.	All 47 countries; WACN and ECSACOP	8-9 April 2 days	English French Portuguese	500 first day 380 second day
Infection Prevention and Control (IPC) for nurses	IPC Dead body handling	West Africa College of Nurses	10-April Morning 3 hours	English French Portuguese	498 nurses from 42 countries across Africa
Clinical management of SARI associated with COVID-19	Asymptomatic infection and transmission; COVID-19 clinical characterization; Triage and hospitalization; Sepsis: Antimicrobial therapy; Treatment of the severely ill patients in facilities; Criteria of discharge and management of convalescent patients; Role of vaccines in the COVID-19 pandemic response; Infection Prevention and Control and handling of bodies	All 47 countries; partners; Universities, WHO country Offices	10-April Afternoon 2.5 hours	English French Portuguese	1200 unique participants from 172 locations in 58 countries

Over the past three months, we have covered other topics such as psychosocial support for nurses caring for COVID-19 patients, how to design an alternate site treatment centre for COVID-19, training of all country incident management teams and epidemic committees on the critical intervention pillars for COVID-19 preparedness and response, and managing ethical issues during the pandemic such as research and development (R and D) of experimental vaccines, therapeutics and diagnostics. As the epidemic matures, this unique model will allow for sharing of individual case studies and best practices from member countries.

### Discussion

There is a high demand for rapid and regular training, information dissemination and education about COVID-19. However, the resources required to meet the needs and demand for training are limited by time, financial and human resources. This challenge has been further compounded by the administrative controls, restriction of travel, and physical distancing interventions implemented by all countries in Africa. COVID-19 has also been associated with another “epidemic of misinformation”. Consequently, there is an urgent need for rapid, evidence-based, and actionable information in Africa. As the SARS-CoV2 virus continues to spread [6], the WHO Africa region is at a critical juncture, needing to quickly support countries to prepare for and respond to COVID-19 while taking into consideration the physical distancing measures, including shelter-in-place and stay at home mandates. This virtual programme based on the ECHO model leverages both existing ECHO platforms in many African countries, and augments preparedness and response efforts to reduce the spread of COVID-19 [12,13].

The first ECHO program in Africa was launched in 2015 by the Namibia Ministry of Health and Social Services (MOHSS) with support from the US Centers for Disease Control (US CDC) and the United States President´s Emergency Plan For AIDS Relief-PEPFAR; focused on HIV capacity building, this virtual community of practice has met weekly for nearly five years and engages doctors, nurses and pharmacists at over 50 hospitals and health care centers across Namibia [15]. Since March 2020, the Namibia MOHSS ECHO network has added COVID-19 training while continuing its HIV capacity building program. More than 15 African countries are implementing a variety of ECHO programs, currently more than 30, focused on topics as diverse as HIV, tuberculosis (TB), laboratory services, cancer, and safe surgery. Several African ECHO programs engage learners and collaborators across multiple countries, including those implemented by the Africa Centres for Disease Prevention and Control (Africa CDC) on the International Health Regulations (IHR) implementation, the Africa Society of Laboratory Medicine on HIV Viral Load Scale Up, and Infection Control Africa Network on the first global ECHO infection prevention and control platform. All these existing networks are being leveraged and expanded to support the COVID-19 response across Africa.

The COVID-19 pandemic and the context it has produced should serve as a catalyst for the rapid expansion of existing networks across Africa and creation of new education and training networks at scale. Low dose, high frequency (LDHF) virtual learning approaches offer the promise of saving time and money while reducing carbon production associated with travel (good for the planet), and there is good evidence that LDHF learning approaches are more effective [16,17]. While the current shift to virtual leaning approaches is being catalyzed by dire necessity, the infrastructure and networks that are being developed have great potential to persist as the post COVID-19 legacy and help positively transform and usher in a new normal for public health and clinical education and capacity building across Africa.

The WHO Africa region COVID-19 virtual learning sessions have initially focused on predominately didactic, overview learning on high priority topics, but rapidly will shift towards increased emphasis on participant driven case-based learning, with participants in the field presenting cases [both patient and system focused] based on questions and challenges of critical importance related to COVID-19. UNM offers instant continuing medical education/continuing professional development (CME/CPD) for participants; this will be connected to anonymous participant session evaluation surveys. Over time, additional topics will be addressed based on learner feedback. Additional cohorts formed around health worker groups and professional organizations will also be identified and supported as needed. For example, WHO has reached out to the West Africa College of Nurses and the East and Central Africa College of Physicians. In addition to live virtual face-to-face learning sessions, each cohort will be supported with virtual asynchronous communication platforms, such as WhatsApp, facilitated by WHO and the professional association secretariat. This will assist in providing real-time updates and increased collaboration within and amongst the networks.

Importantly, the virtual trainings will be used to create communities of practice to build capacity and promote collaboration across all African countries, in the form of training, mentorship, South-to-South collaboration and sharing of best practices. It is our considered view that the challenge of COVID-19 has created an opportunity for WHO, other technical agencies and ministries of health to ensure that all countries are: 1) enabled to provide high-quality, people-centered COVID-19 mitigation interventions, while maintaining comprehensive essential services; and 2) to measure and monitor data on the COVID-19 pandemic to inform policy development.

This WHO Africa region COVID-19 capacity building initiative supported by diverse partners is providing consistent communication, collaboration and education resources for low-dose, high-frequency learning through virtual communities of practice. The programme is leveraging technology to bridge geographic distance during the current lockdowns and focusses on building capacity of participating teams to optimally disseminate and implement appropriate interventions for COVID-19. Additionally, and importantly, the programme is strengthening WHO´s technical leadership and coordination capacity to prepare for and respond to the current COVID-19 pandemic and to future public health emergencies. Moreover, the programme is ensuring equal access and equity in capacity building since all countries (at national and sub-national levels) can access simultaneously and in real time the much-needed information on the COVID-19 pandemic in all the three WHO Africa region official languages.

Finally, the WHO Africa region and all the collaborating partners are working closely with highly qualified consultants to devise an evaluation plan that will measure a variety of program outcomes. WHO and partners will track attendance and administer surveys that invite participants to describe areas of greatest need in their countries and provide on-going feedback on program relevance. The evaluation team will also organize focus groups to further engage in bidirectional learning with the existing workforce and better understand how participation influences outcomes for patients and their providers. The evaluation team will plan and collect data for other outcomes including provider satisfaction, declarative and procedural knowledge gains, changes to practice, and higher-level outcomes as appropriate.

## Conclusion

From the Spanish flu pandemic of 1918, which caused the death of more than 50 million people, to H5N1, H1N1, SARS, MERS, Ebola and now COVID-19, epidemics and pandemics have had devastating social and economic impacts [18-23]. We cannot predict which pathogen will cause the next pandemic, nor where it will occur, nor how dire the effects will be - but if humans and infectious disease pathogens coexist, pandemics will continue to occur. COVID-19 has shown us that we need to be prepared and able to quickly reach all health workers with up-to-date information and guidance to help optimize the public health and clinical response, including information that will both help protect and save the lives of patients and health care workers; one way to achieve this is by using innovative virtual peer-to-peer learning platforms.
